# Social Vulnerability, Frailty and Mortality in Elderly People

**DOI:** 10.1371/journal.pone.0002232

**Published:** 2008-05-21

**Authors:** Melissa K. Andrew, Arnold B. Mitnitski, Kenneth Rockwood

**Affiliations:** 1 Division of Geriatric Medicine, Dalhousie University, Halifax, Nova Scotia, Canada; 2 Department of Medicine, Dalhousie University, Halifax, Nova Scotia, Canada; 3 Department of Mathematics and Statistics, Dalhousie University, Halifax, Nova Scotia, Canada; University of Bern, Switzerland

## Abstract

**Background:**

Social vulnerability is related to the health of elderly people, but its measurement and relationship to frailty are controversial. The aims of the present study were to operationalize social vulnerability according to a deficit accumulation approach, to compare social vulnerability and frailty, and to study social vulnerability in relation to mortality.

**Methods and Findings:**

This is a secondary analysis of community-dwelling elderly people in two cohort studies, the Canadian Study of Health and Aging (CSHA, 1996/7–2001/2; N = 3707) and the National Population Health Survey (NPHS, 1994–2002; N = 2648). Social vulnerability index measures that used self-reported items (23 in NPHS, 40 in CSHA) were constructed. Each measure ranges from 0 (no vulnerability) to 1 (maximum vulnerability). The primary outcome measure was mortality over five (CHSA) or eight (NPHS) years. Associations with age, sex, and frailty (as measured by an analogously constructed frailty index) were also studied. All individuals had some degree of social vulnerability. Women had higher social vulnerability than men, and vulnerability increased with age. Frailty and social vulnerability were moderately correlated. Adjusting for age, sex, and frailty, each additional social ‘deficit’ was associated with an increased odds of mortality (5 years in CSHA, odds ratio = 1.05, 95% confidence interval: 1.02–1.07; 8 years in the NPHS, odds ratio = 1.08, 95% confidence interval: 1.03–1.14). We identified a meaningful survival gradient across quartiles of social vulnerability, and although women had better survival than men, survival for women with high social vulnerability was equivalent to that of men with low vulnerability.

**Conclusions:**

Social vulnerability is reproducibly related to individual frailty/fitness, but distinct from it. Greater social vulnerability is associated with mortality in older adults. Further study on the measurement and operationalization of social vulnerability, and of its relationships to other important health outcomes, is warranted.

## Introduction

As people age and become more vulnerable, their social circumstances particularly impact their health.[1]–[8] Even so, the many descriptions of how social factors, aging and health relate to each other employ various terms. Social inequalities, social environments, sense of life control and coherence, social support, social networks, social engagement, social capital, social cohesion, and socioeconomic status each have been associated with health status.[9]–[11] While the varying terminology reflects different traditions and fields of study, a useful discipline is imposed by aiming for an approach that is feasible, valid, rooted in clinical practice and summarizable for policy-making.

Recent work on the quantification of fitness and frailty might provide a guide to quantifying social vulnerability.[12], [13] A series of studies has shown that health status can be summarized by a deficit accumulation approach, *i.e.* counting the deficits present in an individual.[14]–[16] The underlying idea is that the more deficits (or problems) an individual has (or accumulates), the more vulnerable she or he will be to insults that an individual with fewer deficits might be able to keep at bay. This has proved to be a robust enough approach to yield comparable estimates of the rate of deficit accumulation of health-related deficits – adding about 3 percent of a list of deficits with each increasing year of age – across several surveys,[17] and to demonstrate replicable limits to frailty.[18]

If a series of individual deficits could be combined to estimate not just relative fitness/frailty, but also social vulnerability, the resulting social vulnerability index variable would offer insights into understanding the complex health and social care needs of older adults. Especially as people become very old, “social” and “medical” factors have a complex inter-play that affects important health outcomes and is important for both clinical care and policy-making, but how to consider so many factors has been a challenge. The aims of the present study were to operationalize social vulnerability according to a deficit accumulation approach, to compare social vulnerability and frailty, and to study social vulnerability in relation to mortality.

## Materials and Methods

### Study samples

**Figure 1:**
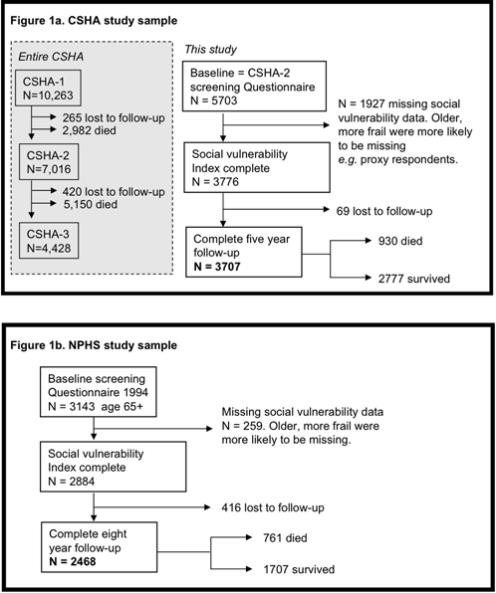
Composition of the Canadian Study of Health and Aging sample (Panel a) and the National Population Health Survey (Panel b).

The Canadian Study of Health and Aging (CSHA) is a representative study of dementia and related conditions in older Canadians (age ≥65 years). Details of the methods are described elsewhere.[19] Briefly, sampling was population-based and representative of English- and French-speaking older Canadians (age ≥65). The sample of 10,263 individuals was clustered within five Canadian regions and stratified by age, with over-sampling of those aged 75 and older. In CSHA-1 (1991–92), a screening interview was conducted with 9,008 community-dwelling participants. Follow-up at 5 years (CSHA-2) and 10 years (CSHA-3) included repeat screening assessments. Of the 10,263 initial CSHA study participants, 9,998 individuals were accounted for at CSHA-2 (of whom 2,982 had died) and 9,578 were accounted for at CSHA-3 (of whom 5,150 had died). Here, baseline data were drawn from the CSHA-2 screening interview, which had included additional information about social factors. As such the study sample comprised community dwelling adults aged 70+ at baseline (Figure 1).

The National Population Health Survey (NPHS) is a panel survey sampling Canadian residents of all ages and administered by Statistics Canada. Survey waves were completed every two years starting in 1994; the most recent available follow-up wave was done in 2002, yielding eight year follow-up. The sampling design included multistage stratification by geographic and socio-economic characteristics and clustering by Census Enumeration Areas.[20] Eight-year follow-up was available for 2468 individuals aged 65+ at baseline who had completed all items in the social vulnerability measure (Figure 1).

### Measures

#### Social vulnerability

Self-report variables relating to social factors that could be considered as deficits were identified separately in the CSHA and the NPHS (Table 1). Deficit selection was guided by two imperatives. First, we aimed to include a broad representation of factors that influence and describe an individual's social circumstance. These factors were based on previous studies which have suggested that they are relevant (*e.g.* social support, social engagement, sense of mastery/control over one's life circumstances).[1]–[4], [8], [21] As part of a holistic description of social vulnerability, socioeconomic status (*e.g.* income adequacy, home ownership – addressing both wealth and housing security – and educational attainment) was also included,[22], [23] as these factors also influence vulnerability to insults with the potential to impact their health status. The two instrumental activities of daily living items included (ability to use the telephone and to get to places outside of walking distance) explicitly relate to an individual's ability to maintain social ties and participate in their community, and were therefore included in the social index. Second, working within the constraints of secondary data analysis, we aimed to make the measure of social vulnerability as sensible and as broadly applicable and comparable between datasets as possible.

**Table 1:** Items aggregated in the social vulnerability index used in each survey.

**Communication to engage in wider community**	
1	Read English or French
2	Write English or French
**Living situation**	
3	Marital status
4	Lives alone
**Social support**	
5	Someone to count on for help or support
6	Feel need more help or support
7	Someone to count on for transportation
8	Feel need more help with transportation
9	Someone to count on for help around the house
10	Feel need more help around the house
11	Someone to count on to listen
12	Feel need more people to talk with
13	Number of people spend time with regularly
14	Feel need to spend more time with friends/family
15	Someone to turn to for advice
16	Feel need more advice about important matters
**Socially oriented Activities of Daily Living**	
17	Telephone use
18	Get to places out of walking distance
**Leisure activities**	
19	How often visit friend or relatives
20	How often work in garden
21	How often golf of play other sports
22	How often go for a walk
23	How often go to clubs, church, community centre
24	How often play cards or other games
**Ryff scales**	
25	Feel empowered, in control of life situation
26	Maintaining close relationships is difficult and frustrating
27	Experience of warm and trusting relationships
28	People would describe me as a giving person
**How do you feel about your life in terms of …**	
29	Family relationships
30	Friendships
31	Housing
32	Finances
33	Neighbourhood
34	Activities
35	Religion
36	Transportation
37	Life generally
**Socio-economic status**	
38	Does income currently satisfy needs
39	Home ownership
40	Education
A) Canadian Study of Health and Aging	
**Communication to engage in wider community**	
1	Can speak English or French
	**Living situation**
2	Marital status
3	Lives alone
**Social support**	
4	Someone to count on for help in crisis
5	Someone to confide in
6	Someone to count on for advice in personal decisions
7	Someone to make you feel loved and cared for
8	Frequency of contact with friends
9	Frequency of contact with relatives
10	Frequency of contact with neighbours
**Social engagement and leisure**	
11	How often participate in groups
12	How often attend religious services
13	Member of voluntary organisations
14	Participation in physical leisure activities (list of 20)
**Empowerment, life control**	
15	Too much is expected of you by others
16	You would like to move but cannot (control/empowerment)
17	Neighbourhood or community is too noisy or polluted
18	You have little control over the things that happen to you
19	Feel that you are a person of worth at least equal to others
20	You take a positive attitude towards yourself
21	How often have people you counted on let you down?
**Socio-economic status**	
22	Not enough money to buy the things you need (income)
23	Educational attainment
B) National Population Health Survey	

Each respondent was assigned a score of 0 if a binary social deficit was absent and 1 if it was present; intermediate values were applied in cases of ordered response categories. For example, an individual scored 1 on the “lives alone” deficit if he/she reported living alone, and 0 if he/she did not. On the “do you ever feel you need more help” deficit, which had three response categories, possible scores were 0 if the answer was “never”, 0.5 for “sometimes” and 1 for “often”. As such, vulnerability on each deficit was mapped to the 0–1 interval. For each individual, a social vulnerability index was constructed using the sum of the deficit scores, yielding a theoretical range of 0–40 in the CSHA and 0–23 in the NPHS. To allow better comparison between the datasets, each with a different number of social deficits, the social vulnerability index was also calculated as a proportion of the total number of deficit items by dividing the sum of deficit scores by the number of deficits considered (40 in the CSHA and 23 in the NPHS), yielding an index with a theoretical range of 0–1.

#### Frailty

Frailty was operationalized analogously to the social vulnerability index, in both the CSHA and NPHS, as described elsewhere.[17], [24] In brief, deficits representing self-reported symptoms, health attitudes, illnesses, and impaired functions (Table S1) were identified and given scores mapping to the 0–1 interval as described above, with a greater score corresponding to worse health status. The social vulnerability and frailty indices were mutually exclusive; no deficits overlapped.

### Statistical analysis

Distributions and properties of both the social vulnerability index and the frailty index were explored using descriptive techniques, including graphs (histograms and scatter/ correlation plots), and descriptive statistics (mean and variance values). ANOVA was used for differences in means, and Chi-square testing for proportions. The characteristics (distributions, means, and ranges) of the frailty and social vulnerability indices were compared and correlations calculated.

Logistic regression modeling was used to determine the association between social vulnerability (explanatory variable) and the primary study outcome of survival at follow-up (five years in CSHA, eight years in NPHS). Survival time was determined by vital status at follow-up and date of death, if the respondent died during the follow-up period, Survival analyses were done using Kaplan Meier curves and Cox proportional hazards regression. All models exploring associations between social vulnerability and survival were adjusted for age, sex, and frailty. Statistical significance of survival differences was assessed using log-rank testing. Proportional sampling weights used where possible to account for sample design.

To investigate the robustness of the composition of the social vulnerability index in respect to individual items, and whether mortality was driven by one or a few of the index's constituent variables, we employed a multi-stage approach. At the design level, we investigated the social vulnerability index in two separate samples, as described. At the instrumental level, we employed two different social vulnerability measures, as also detailed above. At the analytical level, we employed two techniques, each based on repeated re-sampling within the index. Established repeated re-sampling techniques such as “jackknifing” and “bootstrapping” are used to estimate variance and confidence intervals.[25] In most applications, the re-sampling is based on observations, or individuals within the sample. Here, as we have done elsewhere with respect to the frailty index,[26]–[28] we have employed these techniques by applying the re-sampling procedure to a group of variables rather than to a group of observations. The earlier analyses with the frailty index have suggested that a greater number of variables is required to ensure stability in the modeling,[28] so these techniques were applied to the CSHA data, which had a high enough number of variables to yield stable estimates. In the first, a “jackknife by variables” procedure, the social vulnerability index was reconstructed *n* times (where *n* is the number of variables in the index), each time leaving out a different variable, such that the total number of included variables in each reconstruction was *n*-1. In the second, a “bootstrap by variables” procedure, the index was reconstructed 100 times, each time randomly sampling 80 percent of the variables such that on each iteration 20 percent of the constituent variables were randomly left out of the index.[28] For both the “jackknife” and “bootstrap by variables” techniques, associations with survival were tested with each resampled and reconstructed version of the social vulnerability index to assess the impact of leaving out single variables or randomly selected groups of variables from the index.

Statistical analyses were done using STATA 8 and Matlab 7.1 software packages.

## Results

### Descriptive analyses

**Figure 2:**
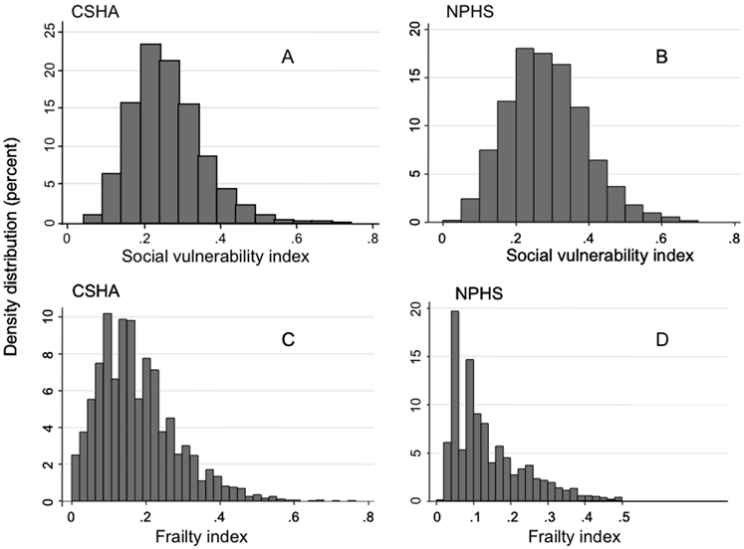
Distributions of social vulnerability: A) Canadian Study of Health and Aging (CSHA), B) National Population Health Survey (NPHS) and frailty: C) CSHA, D) NPHS. While some individuals scored “zero” on the frailty index, no individual was completely free of social vulnerability.

**Figure 3:**
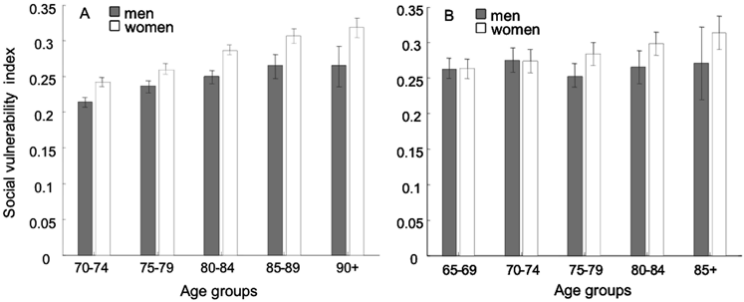
Mean (95% Confidence Interval) social vulnerability in relation to age and sex. Panel A) In the Canadian Study of Health and Aging, social vulnerability increased with age and women had higher index scores than men at all ages. Panel B) In the National Population Health Survey, women showed a trend towards higher scores at older ages.

Mean age was 77.9 (95% CI: 77.8–78.1) in the CSHA and 73.4 years (95% CI: 73.0–73.7) in the NPHS. The samples comprised 60% women in the CSHA and 58% women in the NPHS. 41% of CSHA participants lived alone, compared with 35% in the NPHS. 66% of CSHA participants had less than secondary school education (<12 years of formal schooling); this was true of 52% in the NPHS. While a few items were strongly correlated (*e.g.* in the CSHA, reading correlated strongly with writing (r = 0.60), and marital status correlated with living alone (r = 0.77)), correlation among the items in the social vulnerability indices was generally weak: CSHA median correlation 0.085, IQR = 0.04–0.14. (Statistics Canada confidentiality agreement for data release does not allow the NPHS correlations to be released or published). The distributions of the social vulnerability and frailty indices were similar in the CSHA and NPHS (Figure 2a–d). Median social vulnerability was 0.25 (0.20, 0.31) in the CSHA and 0.28 (IQR 0.21,0.35) in the NPHS. While some people showed no degree of frailty, no individual was completely free of social vulnerability in either dataset. In both samples, social vulnerability increased weakly but significantly with age; women had higher index scores than men at all ages in the CSHA and this trend was present in the NPHS (Figure 3). The social vulnerability and frailty indices were weakly to moderately correlated with each other. The correlations were higher for women than for men (CSHA r = 0.37 for men and 0.47 for women; NPHS r = 0.13 for men and r = 0.24 for women).

### Mortality

Adjusting for age, sex, and frailty, each additional social deficit in the index was associated with an increased odds of death over five years in the CSHA (OR = 1.05, 95% CI: 1.02, 1.07) and eight years in the NPHS (OR = 1.08, 95% CI: 1.03, 1.14). Cox regression modeling yielded similar results: adjusting for age, sex, and frailty, each additional social deficit increased the risk of death by 3% in the CSHA (HR 1.03, 95% CI: 1.01–1.05) and 4% in the NPHS (HR 1.04, 95% CI: 1.01–1.07). Using the index operationalization that scales each index to values between 0 and 1, thereby adjusting for the different number of deficits included in the two indices and allowing direct comparison between the two datasets, the strength of association was similar in the CSHA and NPHS. For this hypothetical comparison of no social vulnerability (index = 0) *vs.* maximal vulnerability (index = 1), adjusting for age, sex, and frailty, maximal vulnerability would confer six times the odds of mortality: OR = 6.22 (95% CI: 2.30, 16.83) in the CSHA and OR = 6.22 (95% CI: 1.82, 21.21) in the NPHS.

**Figure 4:**
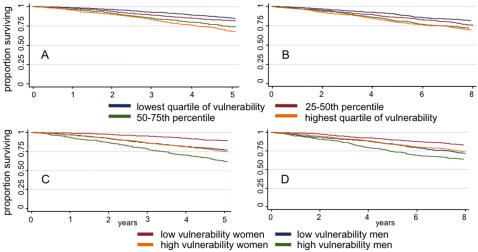
Survival by level of social vulnerability. Panels A (Canadian Study of Health and Aging) and B (National Population Health Survey) show decreasing survival by increasing quartile of social vulnerability. Panels C (CHSA) and D (NPHS) show that although women had better survival than men, survival for women with high social vulnerability was equivalent to that of men with low vulnerability.

Survival decreased progressively in each quartile of increasing social vulnerability (Figure 4a&b). This survival gradient remained statistically significant when adjusted for age and sex (stratified log-rank test *p*<0.001 in both the CSHA the NPHS). Further adjusting for frailty, the survival gradient remained statistically significant in the NPHS (stratified log-rank test *p* = 0.04) but not in the CSHA (*p* = 0.15). Although women had better survival than men, survival for women with high social vulnerability was equivalent to that of men with low vulnerability (Figure 4c&d).

### Re-sampling techniques

**Figure 5:**
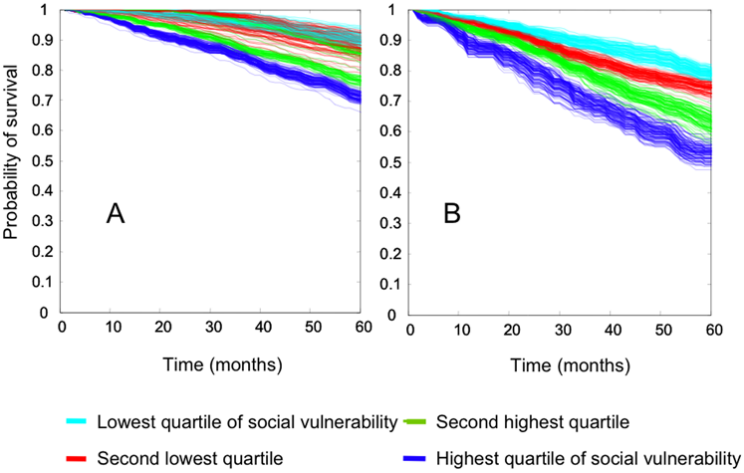
‘Bootstrap by variables’ analyses. Survival curves show 100 replications of 80% re-sampling within the Canadian Study of Health and Aging social vulnerability index. Panel A – women, Panel B – men.

Associations with mortality, adjusted for age, sex, and frailty, remained unchanged as each individual social deficit was left out of the CSHA social vulnerability index in the “jackknife by variables” procedure (Table S2). Survival analysis results using the “bootstrap by variables” technique are shown in Figures 5a&b. The separation between quartiles of social vulnerability remains clear for men despite random omission of 20% of the index variables in each iteration. For women, the separation was clear for the two quartiles indicating the highest social vulnerability, but less so for those with lower vulnerability according to the index.

## Discussion

We used a social vulnerability index to evaluate social factors as they relate to older adults' health. The distribution of social vulnerability was such that no individual was free of social deficits. Social vulnerability increased with age, and women had higher index values than men. Social vulnerability was weakly to moderately correlated with frailty; while the two may be related, they are clearly distinct, particularly since each contributes independently to mortality. Increasing social vulnerability was associated with reduced medium-term survival (5–8 years).

Our findings must be interpreted with caution. Our operationalization of social vulnerability was based on self-report data rather than on objectively defined social factors. Thus it is possible that some individuals over-report and others under-report vulnerability. Further study of distinctions between subjective and objective aspects of social vulnerability is warranted. It is, however, conceivable that older adults' self-perceived vulnerability may be more relevant to their health and quality of life than more objective measures. While we found that social vulnerability increases with age, it was not possible to distinguish between accumulation of deficits with age and the possibility of cohort differences in vulnerability. This would require a different study design (follow-up of different age-based cohorts over time) but warrants further study. In addition, each study had important non-response, and we found that people who did not respond, or who did not have information on social factors were frailer and older. They had higher mortality rates and were more likely to be institutionalized. As both increasing age and increasing frailty are associated with increased social vulnerability, exclusion of these individuals may have led to underestimates of the levels of social vulnerability in the populations of older Canadians represented by the samples, and may have resulted in conservative estimates of associations between social vulnerability and mortality. Little is known about social vulnerability in institutional settings, but given that institutional living would affect social vulnerability in important ways (*e.g.* not living alone, access to social support, networks, and activities), further research is warranted.

We devised tests to address another potential critique of our approach: whether some individual items included in the index drive the identified associations with mortality. If this were the case for one or more variables, an argument might be made it/that they should not be combined in the index. For example, items such as income and education could be treated as separate confounders rather than being included in the index. For this reason, we investigated whether inclusion or exclusion of individual variables (using the “jackknife by variables” technique), and groups of variables (using the “bootstrap by variables technique”) materially affect the analysis results. We have demonstrated that inclusion or exclusion of single variables in the index does not affect the results, and that the same may be true for randomly selected groups of variables (particularly for men), when a sufficient number of variables are included in the index. Of course, the unit of observation is important: for individuals, knowing exactly which deficits are present is likely to be important, but at a population level we find that the number of deficits (rather than the content of these deficits) is more predictive of mortality.

Our findings are consistent with previous research associating various social factors, generally studied one at a time, with health and survival. For example, increased social ties, participation in groups, contact with friends and family, and perceived social support have been associated with survival.[1]–[4] Social disengagement, low participation in leisure activities, and limited social networks have been associated with cognitive decline and dementia[5], [7], [29], [30] and disability.[8] Trust and voluntary sector participation are associated with survival at state and neighbourhood levels.[11], [31] Weak social cohesion has been proposed as an explanation for observed links between poor health and income inequalities[32] and social status inequalities.[21]

The social vulnerability index is a new measure which allows pragmatic quantification of important health information. It appears to be a valid measure, as it predicts mortality and has preserved properties in two independent samples, though further study is warranted to strengthen understanding of its validity and properties. Validation in further independent samples is warranted. *Content and construct validity* are addressed by embedding the index in a theoretical framework[33], [34] and including social factors that have been found to be relevant in characterizing individuals' social situations and to health outcomes. The weak to moderate correlation seen with frailty is evidence for *criterion and convergent validity*, as some relationship between social vulnerability and frailty is reasonable, though the two are distinct measures. The remarkable conservation of the properties of the social vulnerability index approach and associations (albeit of two indices differing in the details of their construction but sharing a common approach and theoretical basis) with health in two different cohorts of older Canadians suggests *generalizability and reproducibility*, though replication in other populations and settings is needed. As the social vulnerability index is a new measure, its reliability (within and between raters, and over time) has yet to be quantified, but is of interest, particularly in considering potential applicability to clinical settings.

The social vulnerability index is an aggregate of items that each have been put forward as reflecting particular aspects of how social factors interact with health. They were not proposed to be combined in the way that we have done, so it is reasonable to ask whether it is fair to combine these many factors into a single index. Two considerations motivated our combining individual factors, even though we recognize that the factors come from different theoretical backgrounds and not all were intended to be combined as we have done. The first is entirely pragmatic. Large numbers of factors are difficult to handle in multivariable models, and require impracticably large sample sizes, especially if interactions are to be modeled. The second motivation for combining factors was so that we could study the properties of the social vulnerability index. In working with the frailty index, we have been struck by the insights that it allows regarding the complexity of frailty. Analyzing the properties of the frailty index has allowed us to employ tools from mathematics which allow us to consider complexity more formally, and not just as a synonym for ‘complicated’. For example, the frailty index appears to accumulate at a characteristic rate across studies (at about 0.03/year on a log scale).[17] Here, accumulation of social vulnerability with age was seen chiefly with women. The frailty index has a characteristic sub maximal limit (about 0.67), *i.e.* people generally do not have more than two thirds of the deficits included in a frailty index – in other words, when the limit has been achieved, no further deficit accumulation is possible, as further deficits would result in death.[18] This is an intriguing observation and we aim to investigate whether there is a maximal limit to how socially vulnerable an individual can become and still survive. Additionally, the frailty index shows reproducible transitions between health states,[35] pointing to additional studies of how individuals transition between levels of social vulnerability – *i.e.* how they accumulate deficits as they move from lower to higher vulnerability.[35]

It is possible that, for example, a principal components analysis might suggest separable domains of social vulnerability. Though such analyses are traditional, they are not without arbitrariness (for example, allowing the operator to specify the number of dimensions to be ‘discovered’), and there are reasons to be skeptical about the approach. It is more instrument-dependent, and thus less generalizable. Many single items that are readily measured in younger people – socioeconomic status in relation to occupation, income and address, for example, are less well measured in people post-retirement, or in neighborhoods in transition.[22] In general, psychometric reductionist techniques consider fewer variables, but lose information. Here we achieved analytical parsimony with just one variable, without losing items that were individually informative. What is more, the index also allows an essentially continuous distribution of risk rather than the artificially small number of risk groups possible with ordinal variables.

Our approach has certain strengths. Several estimates were closely replicable, despite the social vulnerability indexes being constructed differently in the two samples. This suggests that the social vulnerability index has potentially wide applicability: the constituent variables can differ in different settings as long as the basic tenant of including multiple social factors relating to important broad domains is met. The holistic quantification and measurement of social vulnerability has great potential relevance for health and social policy. Being able to identify individuals and groups who are social vulnerable could be useful for prediction of health outcomes as well as for targeting of interventions and design of specialized programs. While it is certainly possible to study the health influence of individual social factors, this “one thing at once” approach is limited, especially for older adults in whom complex sets of social circumstances may exist and interact in different (possibly unpredictable) ways to contribute to vulnerability in an aggregate sense. Even older adults who have a particular deficit (*e.g.* who live alone) would still be differentially susceptible to insults of circumstance (*i.e.* those insults that perturb the delicate balance of assets and deficits, strengths and weaknesses, which has thus allowed them to maintain their heath), depending on their profile of other deficits and strengths.

Several of our findings point to interesting sex differences in social vulnerability. In both the CSHA and the NPHS, women had higher social vulnerability index values than men. One might wonder whether this is due to older age among women, but the finding was independent of age. Correlations with frailty also differed, and were higher for women than for men. Additionally, although women had better survival than men (consistent with many other epidemiological studies), high social vulnerability in women seems to negate this sex benefit, reducing their survival to equal that of less vulnerable men (Figure 4). The index's composition also seems to matter differently between the sexes. For men, survival analyses using re-sampling techniques maintained clear separation into quartiles of social vulnerability, suggesting that for men the specific variables included in the index are not as important as the overall impression of vulnerability. For women, separation was quite clear for the two highest quartiles of social vulnerability, although there was overlap in the survival curves of those less vulnerable. This suggests that for the most socially vulnerable women, the specific individual variables included in the index are less important than they are for those less vulnerable. The reasons for sex differences in the characterization of social vulnerability and in associations with survival are unclear, suggesting a need for further research. Possible contributing factors include sex differences in self-reporting behaviour or coping strategies.

### Conclusions

In two separate samples, we have found that social vulnerability is higher amongst people who are frailer, and that social vulnerability is associated with higher mortality, independent of frailty. Although much work needs to be done in characterizing social vulnerability, clinical and public health services for older people need to recognize that attention to social factors is integral to the provision of care.

## Supporting Information

Table S1 Frailty index constituent variables in a) the Canadian Study of Health and Aging (CSHA) and b) the National Population Health Survey (NPHS).
(0.08 MB DOC)

Table S2 Jackknife by variables results using CSHA data. Excluding individual variables from the index does not affect associations with mortality.
(0.07 MB DOC)
