# Supplementary material for: Social Vulnerability, Frailty and Mortality in Elderly People
Source: PLoS One. 2008 May 21;3(5):e2232. doi: 10.1371/journal.pone.0002232 (PMC2375054; doi:10.1371/journal.pone.0002232)
Supplement: Table S1 — Frailty index constituent variables in a) the Canadian Study of Health and Aging (CSHA) and b) the National Population Health Survey (NPHS). (0.08 MB DOC) [file pone.0002232.s001.doc]

Table S1. Frailty index constituent variables in a) the Canadian Study of Health and Aging (CSHA) and b) the National Population Health Survey (NPHS).

a) CSHA frailty index

|  | **Variable** |
| --- | --- |
| 1 | Eyesight |
| 2 | Hearing |
| 3 | Help to eat |
| 4 | Help to dress and undress |
| 5 | Help to take care of appearance |
| 6 | Help to walk |
| 7 | Help to get in and out of bed |
| 8 | Help to take a bath or shower |
| 9 | Help to go to the toilet |
| 10 | Help in shopping |
| 11 | Help to prepare own meals |
| 12 | Help to do housework |
| 13 | Ability to take medications |
| 14 | Ability to handle own finances |
| 15 | Poor self-assessed health |
| 16 | Hypertension |
| 17 | Heart and circulation problems |
| 18 | Stroke or effects of a stroke |
| 19 | Arthritis or rheumatism |
| 20 | Parkinson’s Disease |
| 21 | Eye trouble |
| 22 | Ear trouble |
| 23 | Dental problems |
| 24 | Chest problems |
| 25 | Stomach problems |
| 26 | Bladder control problems |
| 27 | Bowel control problems |
| 28 | Trouble with feet or ankles |
| 29 | Trouble with skin |
| 30 | Fractures |
| 31 | Frequent trouble with pain |

As previously published[24] but done using a different wave of the CHSA, leaving out items in the social vulnerability index (living alone, telephone use, and ability to get places out of walking distance).

b) NPHS frailty index

|  | **Variable** |
| --- | --- |
| 1 | Activities around home restricted due to health problems |
| 2 | Difficulty with meal preparation |
| 3 | Difficulty with shopping |
| 4 | Difficulty with everyday housework |
| 5 | Difficulty with chores |
| 6 | Difficulty with self care |
| 7 | Difficulty with moving about inside home |
| 8 | Food allergy |
| 9 | Other allergies |
| 10 | Asthma |
| 11 | Arthritis |
| 12 | Back problems excluding arthritis |
| 13 | Hypertension |
| 14 | Migraines |
| 15 | Chronic bronchitis, emphysema |
| 16 | Sinusitis |
| 17 | Diabetes |
| 18 | Epilepsy |
| 19 | Heart disease |
| 20 | Cancer |
| 21 | Stomach trouble or ulcers |
| 22 | Stroke |
| 23 | Urinary incontinence |
| 24 | Alzheimer’s disease, dementia |
| 25 | Cataracts |
| 26 | Glaucoma |
| 27 | Other diseases |
| 28 | Vision problems |
| 29 | Hearing problems |
| 30 | Speech problems |
| 31 | Trouble getting around the neighbourhood |
| 32 | Dexterity |
| 33 | Trouble with thinking to solve everyday problems |
| 34 | Trouble with Pain |
| 35 | Pain severity |
| 36 | Feelings – happiness |

As previously published[17] leaving out items in the social vulnerability index.
