# Supplementary material for: Social Vulnerability, Frailty and Mortality in Elderly People
Source: PLoS One. 2008 May 21;3(5):e2232. doi: 10.1371/journal.pone.0002232 (PMC2375054; doi:10.1371/journal.pone.0002232)
Supplement: Table S2 — Jackknife by variables results using CSHA data. Excluding individual variables from the index does not affect associations with mortality. (0.07 MB DOC) [file pone.0002232.s002.doc]

Table S2. Jackknife by variables results using CSHA data. Excluding individual variables from the index does not affect associations with mortality.

| Index excludes | Odds Ratio | 95% Confidence Interval | P>|z| |
| --- | --- | --- | --- |
|
|
| full index | 1.06 | 1.02 - 1.10 | 0.002 |
| Education | 1.06 | 1.02 - 1.09 | 0.003 |
| Read | 1.06 | 1.02 - 1.10 | 0.001 |
| Write | 1.06 | 1.02 - 1.10 | 0.001 |
| Marital status | 1.06 | 1.03 - 1.10 | 0.001 |
| Lives alone | 1.07 | 1.03 - 1.10 | 0.001 |
| Support - help | 1.06 | 1.02 - 1.10 | 0.002 |
| Feel need more help | 1.06 | 1.02 - 1.10 | 0.001 |
| Support -transportation | 1.06 | 1.02 - 1.10 | 0.002 |
| Feel need more help with transportation | 1.06 | 1.02 - 1.10 | 0.002 |
| Support -chores | 1.07 | 1.03 - 1.11 | 0.001 |
| Feel need more help with chores | 1.06 | 1.02 - 1.10 | 0.002 |
| Support - listen | 1.06 | 1.02 - 1.10 | 0.002 |
| Feel need more people to talk with | 1.06 | 1.02 - 1.10 | 0.002 |
| Support –number of visits | 1.06 | 1.02 - 1.10 | 0.003 |
| Feel need more visits | 1.06 | 1.02 - 1.10 | 0.002 |
| Support - advice | 1.06 | 1.02 - 1.10 | 0.001 |
| Feel need more advice | 1.06 | 1.02 - 1.10 | 0.001 |
| Telephone use | 1.06 | 1.02 - 1.10 | 0.002 |
| Get out of walking distance | 1.06 | 1.02 - 1.09 | 0.003 |
| Visit friends & relatives | 1.06 | 1.02 - 1.10 | 0.002 |
| Gardening | 1.06 | 1.02 - 1.10 | 0.003 |
| Participate in sports | 1.06 | 1.02 - 1.10 | 0.002 |
| Go for a walk | 1.06 | 1.02 - 1.10 | 0.002 |
| Clubs, church | 1.06 | 1.02 - 1.10 | 0.003 |
| Play cards, games | 1.06 | 1.02 - 1.09 | 0.003 |
| Control, empowerment | 1.06 | 1.02 - 1.10 | 0.002 |
| Close relationships | 1.06 | 1.02 - 1.10 | 0.001 |
| Trusting relationships | 1.06 | 1.02 - 1.10 | 0.002 |
| Giving person | 1.06 | 1.02 - 1.10 | 0.002 |
| Relationships | 1.06 | 1.02 - 1.10 | 0.001 |
| Friends | 1.06 | 1.02 - 1.10 | 0.002 |
| Housing situation | 1.06 | 1.02 - 1.10 | 0.001 |
| Finances | 1.06 | 1.02 - 1.10 | 0.001 |
| Neighbourhood | 1.06 | 1.02 - 1.10 | 0.001 |
| Active | 1.06 | 1.02 - 1.10 | 0.002 |
| Religion | 1.06 | 1.02 - 1.10 | 0.001 |
| Transport | 1.06 | 1.02 - 1.10 | 0.002 |
| Life generally | 1.06 | 1.02 - 1.10 | 0.002 |
| Income sufficient | 1.06 | 1.02 - 1.10 | 0.001 |
| Home ownership | 1.06 | 1.02 - 1.10 | 0.001 |
